# Parotid Gland Mass as the First Manifestation of Recurrent Metastatic Breast Carcinoma: Diagnostic Pitfalls and Therapeutic Considerations in Oral-Maxillofacial Care

**DOI:** 10.3390/curroncol32110634

**Published:** 2025-11-13

**Authors:** Esteban Raúl Mar-Uribe, Miguel Angel Noyola-Frías, Oscar Arturo Benítez-Cárdenas, Elhi Manuel Torres-Hernández, Adalberto Mosqueda-Taylor, Raquel Sánchez-Gutiérrez, Sofía Bernal-Silva, Andreu Comas-García, Francisco Javier Aguilar-Zapata, Ricardo Martínez-Rider, Marlen Vitales-Noyola

**Affiliations:** 1 Service of Oral and Maxillofacial Surgery, Regional High Specialty Hospital Dr. “Ignacio Morones Prieto”, San Luis Potosí 78290, Mexico; ermu27@gmail.com (E.R.M.-U.); manf001@uaslp.mx (M.A.N.-F.); oscar.benitez@uaslp.mx (O.A.B.-C.); manuel.torres@uaslp.mx (E.M.T.-H.); 2 Department of Oral Surgery, School of Dentistry, Autonomous University of San Luis Potosí, San Luis Potosí 78290, Mexico; raquel.sanchez@uaslp.mx (R.S.-G.); francisco.aguilar@uaslp.mx (F.J.A.-Z.); rmrider@uaslp.mx (R.M.-R.); 3 Department of Health Attention, Metropolitan Autonomous University Xochimilco, Mexico City 04960, Mexico; mosqueda@correo.xoc.uam.mx; 4 Department of Microbiology, School of Medicine, Autonomous University of San Luis Potosí, San Luis Potosí 78290, Mexico; sofia.bernal@uaslp.mx (S.B.-S.); andreu.comas@uaslp.mx (A.C.-G.); 5 School of Medicine, Universidad Cuauhtémoc San Luis Potosí, San Luis Potosí 78290, Mexico; 6 Endodontics Postgraduate Program, Faculty of Dentistry, Autonomous University of San Luis Potosí, San Luis Potosí 78290, Mexico

**Keywords:** parotid gland, tumor, breast cancer, metastatic breast carcinoma, maxillofacial surgery

## Abstract

Breast cancer rarely metastasizes to the parotid gland. Early recognition in patients with a history of malignancy is critical for timely diagnosis and treatment. We report the case of a 60-year-old female who presented with a two-month history of a left periauricular mass, 18 months after completing treatment for breast carcinoma. Despite the patient’s oncologic history, initial evaluation by our maxillofacial surgery service showed no evidence of distant metastasis, and we initially ruled out metastatic disease. Clinical evaluation, contrast-enhanced computed tomography (CT), fine-needle aspiration cytology (FNAC), PET-CT, and histopathological analysis were performed. Given the persistent and progressive nature of the mass, surgical excision was undertaken to obtain a definitive diagnosis and provide local control. Immunohistochemical analysis of the resected mass and adjacent node confirmed metastatic breast carcinoma infiltrating the parotid parenchyma and an intra-parotid lymph node, with strong positivity for progesterone receptor (PR) and carcinoembryonic antigen (CEA). Unfortunately, several months later, the patient developed pulmonary metastases and subsequently died.

## Simple Summary

Breast cancer metastasis to the parotid gland is extremely rare and can easily be misinterpreted as a primary salivary gland tumor. In this report, we describe a 60-year-old woman who developed a parotid mass 18 months after completing breast cancer treatment. Despite her oncologic history, the initial clinical and imaging findings were inconclusive. Comprehensive evaluation including CT, PET-CT, and histopathological analysis confirmed metastatic breast carcinoma infiltrating the parotid gland. This case underscores the importance of considering metastatic disease in the differential diagnosis of parotid masses, particularly in patients with a prior history of breast carcinoma, to ensure timely recognition, accurate diagnosis, and appropriate multidisciplinary management.

## 1. Introduction

Breast cancer is one of the most prevalent malignancies and a leading cause of cancer-related mortality worldwide, with approximately 2.3 million new cases diagnosed, according to the World Health Organization (WHO) [1,2,3], and is classified into four molecular subtypes based on estrogen receptor (ER), progesterone receptor (PR), human epidermal growth factor receptor 2 (HER2), and the proliferation marker Ki-67. These subtypes include luminal A (ER+ and/or PR+, HER2−, low Ki-67), luminal B (ER+ and/or PR+, HER2+ and/or high Ki-67), HER2-positive (ER−, PR−, and HER2+), and triple-negative (ER−, PR−, and HER2−). Each subtype significantly influences prognosis and guides treatment strategies [4,5,6,7,8,9]. Metastasis is a major determinant of prognosis in breast cancer, with the bone, liver, lung, and brain being the most common sites of dissemination [10,11,12]. Metastasis to the salivary glands, particularly the parotid gland, is exceedingly rare, accounting for only 5% of malignant salivary gland tumors [13]. While most metastases originate from primary head and neck tumors, 10–20% arise from infraclavicular tumors, such as the bronchi, breast, and kidneys [14,15]. Because of its rarity, parotid gland metastasis often mimics salivary gland tumors, posing diagnostic challenges and delaying appropriate management. Management of parotid metastases does not significantly impact overall survival. Cases with metastases limited to the parotid gland and bone report a mean survival of 4.5 years, while those with additional visceral metastases average 1 to 2 years. Furthermore, the interval between primary diagnosis and parotid involvement appears to have no prognostic relevance [16]. Here, we show an interesting and rare case; there was initially no evidence of metastasis, and fine-needle aspiration biopsy did not reveal malignancy. Surgery was pursued under the suspicion of a benign tumor unrelated to the patient’s history of breast cancer, as the PET-CT performed at that time showed no metastatic disease. However, months later, the cancer recurred with metastatic involvement. Despite these considerations, we present this case of parotid gland metastasis from breast cancer in a 60-year-old female to contribute to the limited available literature. Surgical excision was chosen as part of a multidisciplinary strategy aimed at local control, symptom relief, and histopathological confirmation. This case highlights the importance of maintaining a high index of suspicion for metastatic disease in patients with a history of malignancy and underscores the value of individualized clinical decision-making in complex scenarios.

## 2. Case Presentation

**Figure 1:**
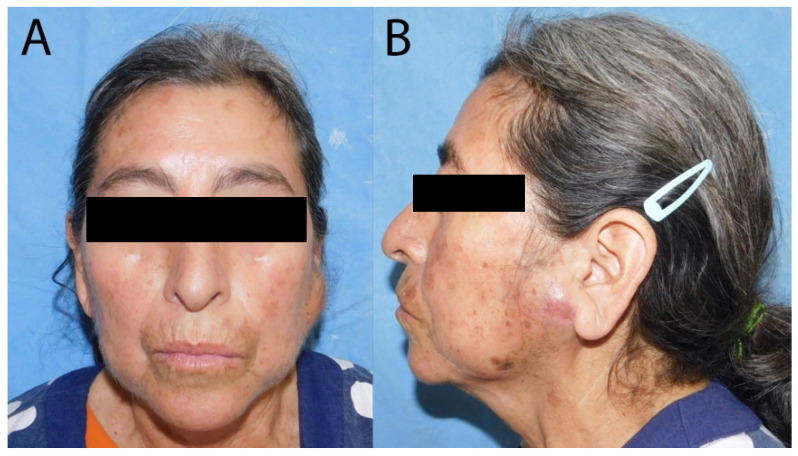
Photographs of the patient before the surgery. (**A**) The frontal view of the patient shows asymmetry due to left parotid swelling; (**B**) the left lateral view demonstrates preauricular swelling accompanied by changes in skin coloration.

A 60-year-old woman presented to the Oral and Maxillofacial Surgery Service at the emergency department with a two-months history of progressive swelling in the left parotid region. Clinical examination revealed a well-circumscribed, erythematous, indurated, and mobile lesion, causing a slight elevation of the left earlobe. There was no warmth to palpation, and the patient reported no pain or neuromotor/neurosensory disturbances, without facial paralysis or enlarged neck nodes. Intraoral inspection revealed standard mouth opening and adequate salivary flow from the left Stenon´s duct. No lymphadenopathy was observed in the cervical supraclavicular regions (Figure 1). Her historical medical records included a right-sided breast carcinoma of no special type (NST), previously referred to as invasive ductal carcinoma, diagnosed two years earlier; the TNM classification [17] was obtained. Tis: Evidence of carcinoma in situ (right breast, carcinoma of no special type, NST). N1: regional lymph node metastasis. Mx: Distant metastasis cannot be assessed. She had undergone a right radical mastectomy followed by 25 sessions of radiotherapy, six cycles of chemotherapy, and maintenance with tamoxifen. The patient had been in remission for 18 months before the current presentation. The patient had no history of other chronic-degenerative diseases or any additional pathological conditions at the time of evaluation. Due to the patient’s history of breast cancer, she was referred to the oncology department, where she was also evaluated by gynecology, internal medicine, and cardiology. Multiple diagnostic tests were requested, including a chest X-ray and a PET-CT scan, among others. These studies did not reveal any clinically significant findings related to the current condition. Therefore, she was referred again to the maxillofacial surgery department for evaluation and management of the current condition.

**Figure 2:**
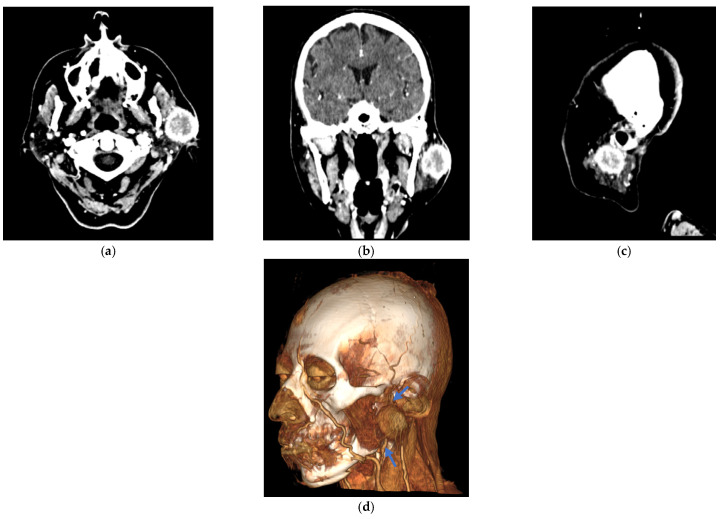
Contrast-enhanced computed tomography (CT) images of the facial mass. A well-defined 30 × 30 mm lesion is highlighted in the left periauricular region of the patient with breast cancer metastasis. (**a**) The axial section shows the lesion in the left periauricular area. (**b**) Frontal plane, showing the lesion. (**c**) The sagittal section confirms the extent of the lesion in the left periauricular region. (**d**) A lateral three-dimensional CT angiogram was obtained using the volume rendering technique (VRT), highlighting the lesion. Blue arrows indicate the retromandibular vein, which serves as the key anatomical landmark for identifying the facial nerve tract within the parotid gland.

Imaging studies, including a head and neck computed tomography scan (CT) with contrast, revealed a 30 × 30 mm well-defined mixed-density nodule in the left parotid gland, showing intense contrast enhancement (Figure 2). Fine-needle aspiration cytology (FNAC) of the lesion was performed and cytologic results revealed chronic inflammatory changes, with no evidence of malignancy, suspected in benign tumor as pleomorphic adenoma. Standard laboratory tests (complete blood count, coagulation profile, and serum chemistry) were within normal limits.

The patient was admitted to surgical management three weeks after the initial evaluation. Using a periauricular incision, the surgeon performed a left superficial parotidectomy under balanced general anesthesia and standard antibiotic prophylaxis. The surgeons carefully identified and preserved the main trunk of the facial nerve. After securing the airway with a No. 7.5 orotracheal tube, asepsis and antisepsis were performed, followed by a skin incision using the modified Blair approach. A superficial parotidectomy was performed with preservation of the facial nerve. Hemostasis was achieved; a Blake drain was placed; and the wound was closed in layers, deep with absorbable sutures and skin with non-absorbable sutures. The patient had an uneventful recovery and was discharged 24 h later with ibuprofen and clindamycin.

Follow-up visits were performed on day three post-discharge and one week later, when the Blake drain was removed. Healing progressed appropriately, with no signs of infection and minimal inflammation.

**Figure 3:**
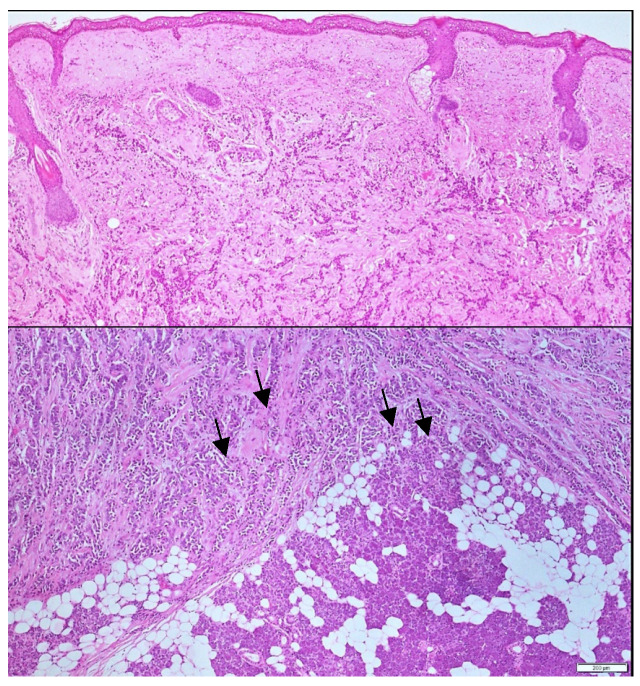
Histological analysis at 10× and 20× magnification. Hematoxylin and eosin sections obtained via incisional biopsy reveal clusters of tumoral cells infiltrating the salivary gland. Black arrows highlight infiltrating metastatic breast carcinoma within the parotid parenchyma.

**Figure 4:**
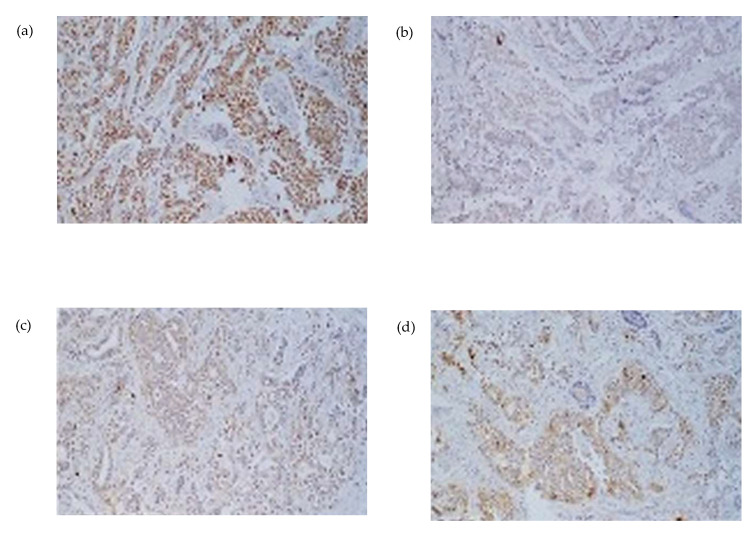
Immunohistochemical staining at 20× magnification from parotidectomy specimen (**a**) progesterone asymmetry due to swelling in the left parotid region receptor (PR), (**b**) Estrogen receptor (RE), (**c**) HER2/neu, and (**d**) carcinoembryonic antigen (CEA). The tumor exhibits strong positivity for PR and CEA in a core needle biopsy, consistent with metastatic involvement.

**Figure 5:**
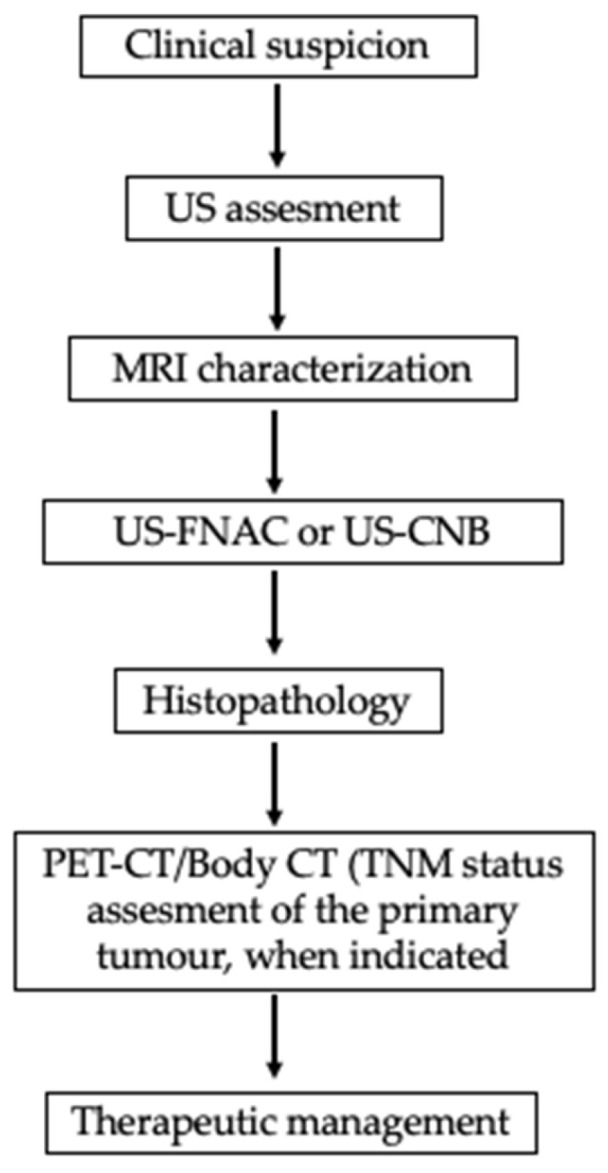
Evaluation of parotid mass in a patient with history of primary malignancy. FDG PET useful when SUV uptake values are high and display the primary and metastatic sites (namely parotid gland). FDG-PET = Positron Emission Tomography with Fluorodeoxyglucose.

The surgical specimen, including the superficial lobe of the parotid gland, was sent for histopathological examination. Microscopic examination showed a metastatic tumor infiltrating both the parotid gland parenchyma and intra-parotid lymph nodes, due to this notable finding, an oral pathologist was consulted. The histological slides were sent to another city for further evaluation to obtain a more accurate diagnosis (Figure 3 and Figure 4), neck nodal level: Level VIII (parotid) per the modified Robbins classification [18]. Immunohistochemistry (IHC) staining was performed to assess ER, PR, HER2, and carcinoembryonic antigen (CEA). Tumor immunostaining showed strong positivity for PR (++90%) and CEA (++90%), consistent with metastatic breast carcinoma origin. Complete data about parotid tumor are showed in next: Pathologic TNM (Histologic) pTis: Carcinoma in situ of the breast, pN0: No regional lymph node metastasis histologically, pNx (SLN): Sentinel lymph node could not be assessed, pN0: No regional lymph node metastasis histologically; no examination of isolated tumor cells (ITCs), pMx: distant metastasis cannot be evaluated microscopically, L0: no lymphatic invasion, V0: no venous invasion. Residual Tumor, R1: microscopic residual tumor present. Histologic Grade, G1: well-differentiated. Postoperatively, the patient was discharged of service of maxillofacial surgery with scheduled follow-up appointments, with no clinical signs of recurrence in the parotid region, but with instructions for oncological evaluation. Five months later, the patient experienced a recurrence of breast cancer with pulmonary metastases and, unfortunately, passed away. Additional a timeline was added (Table 1) and a diagram of clinical suspicion (Figure 5).

**Table 1:** Patient timeline.

Time	Event	Key Details
Year −2	Primary right breast cancer	Carcinoma NST, initial TNM recorded as Tis N0 Mx. Treatment: right radical mastectomy, 25 radiotherapy sessions, 6 chemotherapy courses and tamoxifen maintenance. Remission for 18 months.
T0 (cytology FNAC)	Left preauricular mass (2-month history)	Well-defined, erythematous, firm, mobile lesion, no facial nerve palsy, no cervical lymphadenopathy. Stensen’s duct patent. Full oncologic work-up requested. FNAC of the lesion Chronic inflammatory changes, no malignancy; pleomorphic adenoma suspected
T0 (imaging)	Contrast-enhanced head/neck CT	Well-defined nodule 30 × 30 mm in the left parotid with avid enhancement.
T0 (cytology)	FNAB of the lesion	Chronic inflammatory changes, no malignancy; pleomorphic adenoma suspected.
T0 (systemic staging)	Oncology consult and studies	Chest X-ray and PET/CT negative for metastatic disease at that time.
T0 + 3 weeks	Surgery	Left superficial parotidectomy (Blair approach), facial nerve preserved; hemostasis achieved, Blake drain placed. Discharged at 24 h with ibuprofen and clindamycin.
T0 + 3 days/+1 week	Postoperative follow-up	Favorable course, drain removed, no signs of infection or local recurrence.
Post-surgery (pathology)	Definitive diagnosis	Metastatic breast carcinoma infiltrating parotid parenchyma and an intraparotid lymph node (level VIII). IHC: PR positive (90%), CEA positive (90%); ER and HER2 negative.
T0 + 5 months	Systemic progression and outcome	Development of pulmonary metastases, death from complications of advanced disease.

## 3. Discussion

Metastases to the parotid gland are extremely rare and often originate from distant primary tumors. Among these, breast cancer has been identified as a potential but uncommon source, since this kind of cancer most commonly metastasizes to the bone, liver, and brain; metastasis to the parotid gland is exceedingly rare, with the first case reported by Meher-Homji et al. in 1967 [19]. Most metastatic parotid lesions arise from head-and-neck primaries (e.g., cutaneous squamous cell carcinoma and melanoma) via lymphatic spread, a pattern more common in older men [20]. For infraclavicular primaries such as breast cancer, two biologically plausible routes should be considered: (i) hematogenous parenchymal seeding and (ii) retrograde lymphatic “skip” metastasis to an intra-parotid lymph node. Notably, the parotid is the only major salivary gland that harbors normal intra-glandular lymph nodes, making a nodal pathway anatomically feasible [21,22]. Hematogenous spread is suggested when imaging/histology show infiltrative tumor within parotid parenchyma without a dominant nodal architecture, whereas a lesion centered on an intra-parotid node, particularly with extracapsular spread supports a lymphatic route. In our patient, hematoxylin-and-eosin sections from an incisional biopsy demonstrated clusters of metastatic carcinoma infiltrating the salivary parenchyma with no recognizable nodal architecture in the sampled tissue; extracapsular spread could not be definitively assessed on the limited biopsy. Taken together, the histologic pattern favors hematogenous parenchymal seeding over retrograde lymphatic “skip” metastasis, although the dissemination route cannot be established with absolute certainty.

A multimodal assessment is essential for parotid swelling of unclear origin, particularly in patients with known primary tumors. CT or MRI helps define lesion characteristics and detect lymph node involvement. Fine-needle aspiration cytology (FNAC) provides a minimally invasive means of obtaining cytological samples; however, its diagnostic accuracy for metastatic lesions can sometimes be limited. When FNAB yields indeterminate or nondiagnostic results, a core-needle biopsy or excisional biopsy may be necessary for a definitive diagnosis. Fine-needle aspiration cytology (FNAC) and ultrasound-guided core needle biopsy (CNB) are valuable diagnostic tools for evaluating parotid gland lesions, offering high sensitivity and specificity while minimizing the need for invasive surgery [23]. In the present case, FNAC was initially performed; however, cytological findings were inconclusive and did not allow for definitive characterization of the lesion. Given the persistent clinical suspicion of malignancy and the superficial location of the tumor in the parotid gland, the multidisciplinary team opted for surgical excision to obtain a comprehensive histopathological diagnosis. Nonetheless, we recognize that US-guided CNB represents a reliable and minimally invasive alternative that should be considered in similar future cases.

Positron emission tomography–computed tomography (PET/CT) is an excellent modality for detecting metastases and was performed in this case. At the time of imaging, no metastatic disease was identified; however, several months later, the patient developed pulmonary metastases and, unfortunately, died from complications of advanced disease. Recent studies highlight the complementary value of multimodal imaging in salivary gland tumor assessment. Kong et al. underscored the diagnostic accuracy of ultrasound, CT, MRI, and PET/CT in distinguishing benign from malignant lesions [24]. Jiang et al. emphasized the role of ^18^F-FDG PET/CT in detecting primary and metastatic sites through high SUV uptake [25], while Kim et al. illustrated MRI’s superior soft-tissue resolution for local staging [26]. Together, these findings support a structured diagnostic approach combining functional and anatomical imaging to optimize management decisions. Pathologically, the differential diagnosis for a parotid mass includes primary salivary gland neoplasms, such as mucoepidermoid carcinoma, acinic cell carcinoma, and salivary duct carcinoma, as well as benign entities like pleomorphic adenoma. In this case, cytopathologic examination revealed inflammatory material without evidence of malignancy and no features suggestive of a reactive or infectious process. These findings were critical in distinguishing the lesion from other potential causes of a parotid mass.

Definitive diagnosis depends on a histopathological analysis of the resected specimen. In this case, the tumor’s morphological features, positive immunohistochemical staining for progesterone receptor (PR) and carcinoembryonic antigen (CEA), and negative estrogen receptor (ER) and human epidermal growth factor receptor 2 (HER2) are characteristic. Some studies suggest that negative ER together with intense CEA favors the diagnosis of salivary duct carcinoma over breast cancer [27,28]. However, the case presented showed higher expression of PR and CEA while ER was lower, confirming the diagnosis of metastatic breast carcinoma and suggesting a hormone-responsive phenotype, providing valuable information for therapeutic decision-making. Such immune profiles are critical for distinguishing metastatic lesions from primary salivary gland neoplasms or other metastatic pathologies. Other immunohistochemical markers commonly used to confirm breast origin, such as CK7, GATA3, or GCDFP-15, were not reported in the available pathology records, representing a limitation in the diagnostic confirmation.

Immunocytochemistry (ICC) applied to ultrasound-guided fine-needle aspiration cytology (US-FNAC) can increase diagnostic accuracy when cellular yield is adequate [15]. The differential includes salivary duct carcinoma, which shares histologic features with breast ductal carcinoma due to their exocrine origin; nevertheless, primary parotid tumors typically show weak, patchy ER/PR expression, whereas strong, diffuse positivity favors a breast origin. In our case, strong PR positivity (+++) supports this interpretation. Whenever feasible, a targeted IHC/ICC panel (ER, PR, HER2 ± GATA3, mammaglobin, and GCDFP-15) helps confirm lineage and guide therapeutic decision-making.

Although the initial FDG PET/CT was negative, this finding could be explained by small-volume disease (partial-volume effect), low-glycolytic phenotypes (e.g., invasive lobular carcinoma), recent systemic treatments, or other technical factors (glycemic control, acquisition timing, motion). A dedicated head-and-neck reevaluation with documented SUVmax was not feasible due to the patient’s death; therefore, the PET negativity should be interpreted cautiously and in the context of the contrast-enhanced CT and histopathology. This represents a limitation of the case and underscores the need to integrate anatomic imaging with tissue sampling (FNAC/CNB) when FDG avidity is discordant.

The treatment strategy for metastatic breast carcinoma in the parotid gland depends on the extent of the disease, tumor biology, and the patient’s overall condition (28). Surgical excision, either through superficial or total parotidectomy, may be indicated to relieve symptoms, facilitate diagnosis, and control local disease. In the case presented, fine-needle aspiration cytology (FNAC) did not reveal malignancy, and oncological studies, including chest X-ray and PET-C, showed no evidence of metastasis in target organs. In our case, fine-needle aspiration cytology (FNAC) was not diagnostic. In our case, it is possible that the FNAC was performed too superficially, which may have prevented the detection of metastatic cells that were indeed present. Instead, only inflammatory changes were observed.

A benign parotid neoplasm was initially suspected, which justified proceeding with surgery; this enabled a definitive diagnosis by histopathology. Despite available treatments, parotid surgery has not been shown to improve survival in the metastatic setting, and parotid involvement carries a poor prognosis, with a five-year survival of only 10% [29,30]. Disease control in metastatic breast cancer relies primarily on systemic therapy–chemotherapy, endocrine therapy, immunotherapy, or HER2-targeted therapy when indicated. Prior studies conclude that radical parotid surgery does not extend overall survival; most interventions for parotid metastases are palliative [31]. In the present case, despite aggressive local management and previous breast-cancer treatment, pulmonary metastases led to death within five months. Although parotid metastases from breast cancer have been reported, the number of documented cases remains small [14,32]. In a retrospective analysis, Guo et al. [33] highlighted the poor prognosis and the limited effect of surgery on overall survival, underscoring the importance of early systemic staging and palliative planning in advanced disease. Clinicians should maintain a high index of suspicion for metastasis when evaluating a new parotid mass in patients with a significant cancer history, as prompt recognition can inform decisions about additional systemic therapy or palliative measures.

Additionally, we have included a table summarizing data from other published studies, highlighting the clinical context of reported cases in the literature. This comparative overview provides a broader perspective and supports the relevance of our findings (Table 2) [34,35,36,37,38,39,40,41,42]; in addition, we have included a table showing the most frequent secondary malignancies of the parotid gland (Table 3) [43].

This rare case underscores the diagnostic complexity of parotid gland metastases. Because clinical and radiologic features often overlap with benign and primary parotid tumors, early recognition is difficult and requires a high index of suspicion, particularly in patients with a prior history of malignancy. When imaging and cytology are inconclusive, US-guided CNB or excisional biopsy with histopathology (and, when indicated, immunohistochemistry) remains the definitive step to secure the diagnosis and guide treatment planning. While the present report offers useful clinical insight, its single-case design limits generalizability. Prospective studies and larger multicenter series are needed to refine diagnostic algorithms, compare therapeutic strategies, and standardize follow-up for metastatic disease involving the parotid gland.

## 4. Conclusions

Metastatic breast cancer to the parotid gland is exceptionally rare, accounting for 1.2% of malignant parotid nodules (54 published cases), and the prognosis is unfavorable in major of cases (mean survival 3 months). When searching for the primary site, first, evaluate the head and neck, and cutaneous origins; then, melanoma; and subsequently, infraclavicular primaries (breast, kidney, etc.). Breast cancer should be the first extra-head and neck primary to be ruled out in female patients. US-guided FNAC performed by an experienced operator, together with ICC/IHC (ER, PR +++), and even CNB in superficial parotid tumors, or rarely surgery, should lead to full final diagnosis. This case highlights the importance of close monitoring and keeping an open mind to consider parotid metastasis in patients with a history of cancer.

## Figures and Tables

**Table 2:** Clinical and pathological summary of breast cancer metastases to salivary glands.

Author, Year and Ref	Laterality of Major Salivary Gland (MSG), Parotid (PG), Submandibular Gland (SMG), R Right, L Left	Breast Primary Histology	Biological Subtype/Markers	Breast TNM at Diagnosis	PET-CT, CT, MRI	Therapy	Survival Months
Ando K. 2011 [34]	PG, L	Invasive lobular carcinoma (primary)	HER2+, ER−, PR−	Stage IV (cT1N3M1)	PG SUV = 9.8	Left mastectomy with axillary clearance surgery, chemotherapy, partial parotidectomy	N/A
Salman L. 2025 [35]	MSG, R	Adenocarcinoma with metastatic carcinoma from a breast primary	GATA3+, CK7+, ER+, HER2−	Stage III	Additional bony metastatic disease	Mastectomy, axillary node dissection, and chemotherapy. Palliative treatment	N/A
Duncan M 2015 [36]	PG, bilateral	Adenocarcinoma of breast	ER+	N/A	N/A	N/A	N/A
Dangore-Khasbage. 2009 [37]	PG, L	Primary: right breast carcinoma	N/A	N/A	N/A	N/A	N/A
Kollias J. 1997 [38]	PG n = 57 24 patients ipsilateral PG, 18 cases contralateral or bilateral PG	Predominant pathological subtype: invasive ductal carcinoma	ER+, PR+, HER2+, GATA3+, Ki67+, FISH−	T2N1M0 = Stage IIB	Multiple bone metastases	21 cases parotidectomy, 9 superficial parotidectomy, 21chemotherapy and 25 radiotherapy	6 months
Ben Dhia S. 2020 [39]	PG, bilateral	Invasive ductal carcinoma (primary)	ER+, PR+, HER2−	pT4N1 = stage IIIA	Metastatic skin nodules and multiple bone metastases	Palliative chemotherapy and hemostatic radiotherapy	5 months
Jung H.K. 2021 [40]	PG, L	Invasive ductal carcinoma, NST	HER2+, ER−, PR−, AR−	pT4N3 = stage IIIC	Right axillary and left neck lymph node, liver, brain, bone, and skin metastases	Radical mastectomy, chemotherapy, radiation therapy, and trastuzumab	2 months
Jakharia-Shah A. 2019 [41]	PG, L	Invasive ductal carcinoma + DCIS (primary)	ER+, HER2+	T2N0M0 = stage IIA	No significant findings	Radiotherapy, chemotherapy, left parotidectomy	N/A
Burgess S.A. 2015 [42]	PG, R	Invasive ductal carcinoma of the right breast	CK7+, ER+, PR+, AR+	N/A	8mm PG irregular nodule	Palliative treatment	N/A

TNM = Tumor–Node–Metastasis, ER = estrogen receptor, PR = progesterone receptor, HER2 = human epidermal growth factor receptor 2, Ki-67 = proliferation index, CK7=cytokeratin 7, N/A = not applicable.

**Table 3:** Main etiologies of malignant parotid tumors according to Mayer et al., 2021 [43], showing the predominance of cutaneous squamous cell carcinoma metastases, followed by lymphomas, melanoma, and other less frequent entities.

Type of Secondary Malignancy	Frequency (%)	Description/Notes
Cutaneous squamous cell carcinoma (SCC) metastases	35.4	Most frequent secondary malignancy infiltrating the parotid gland
Lymphomas	14.0	Second most common; includes non-Hodgkin types
Malignant melanoma (MM) metastases	6.1	Often from facial or scalp primary lesions
Carcinoma of unknown primary (CUP syndrome)	3.7	Metastatic parotid involvement with undetermined primary origin
Mucosal squamous cell carcinoma metastases	3.0	Secondary to mucosal head and neck sites
Merkel cell carcinoma metastases	2.4	Neuroendocrine skin carcinoma with parotid spread
Merkel cell carcinoma infiltration	1.8	Direct invasion into parotid tissue
Breast carcinoma metastases	1.2	Rare; usually from advanced systemic disease
Renal cell carcinoma metastases	1.2	Rare metastatic spread to parotid gland
Basal cell carcinoma infiltration	<1	Very uncommon; single reported case
Langerhans cell histiocytosis	<1	Rare histiocytic lesion involving parotid tissue
Sarcoma	<1	Exceptional occurrence
Sinonasal adenocarcinoma metastasis	<1	Extremely rare presentation

## Data Availability

The raw data supporting the conclusions of this article will be made available by the corresponding authors on request.
